# Long-Term Testosterone Shows Cardiovascular Safety in Men With Testosterone Deficiency in Electronic Health Records

**DOI:** 10.1210/jendso/bvaf074

**Published:** 2025-05-07

**Authors:** Yilu Lin, Shaveta Gupta, Lizheng Shi, Franck Mauvais-Jarvis, Vivian Fonseca

**Affiliations:** Department of Health Policy and Management, School of Public Health and Tropical Medicine, Tulane University, New Orleans, LA 70112, USA; Department of Medicine, Section of Endocrinology, School of Medicine, Tulane University, New Orleans, LA 70112, USA; Department of Health Policy and Management, School of Public Health and Tropical Medicine, Tulane University, New Orleans, LA 70112, USA; Department of Medicine, Section of Endocrinology, School of Medicine, Tulane University, New Orleans, LA 70112, USA; Southeast Louisiana VA Healthcare System, Hormone Therapy Clinic, New Orleans, LA 70119, USA; Department of Medicine, Section of Endocrinology, School of Medicine, Tulane University, New Orleans, LA 70112, USA; Southeast Louisiana VA Healthcare System, Endocrine Clinic, New Orleans, LA 70119, USA

**Keywords:** testosterone therapy, cardiovascular safety, real-world evidence

## Abstract

**Objective:**

Our objective is to examine the association between cardiovascular (CV) safety and long-term testosterone therapy (TTh) in men with testosterone deficiency (TD) in real-world practice.

**Method:**

We extracted the electronic health records of 2683 adult men with TD from 3 healthcare systems from January 1, 2012, to June 30, 2023. We matched TTh and non-TTh groups in a 1:1 ratio based on age, race, Charlson Comorbidity Index, and serum testosterone level via propensity score. We used intent-to-treat analysis using Kaplan-Meier curves and Cox regressions to examine CV risk for major adverse cardiovascular events (MACE). We also explored the impact of TTh on diabetes and hyperlipidemia development and progression. We compared 928 TTh patients to 928 untreated patients with a median follow-up of 3 years for both groups.

**Results:**

After matching, body mass index, diastolic blood pressure, hyperlipidemia, hypertension, depression, and anxiety were statistically significant different between treatment and control cohorts. The log-rank test for the cumulative MACE incidence was comparable (*P* > .05). There were no statistically significant associations between TTh use and CV risk hazard ratios (HRs) in the univariate Cox regression (HR [95% CI]: 1.01 [0.75-1.36]) and Cox regressions adjusted by the preexisting MACE (HR [95% CI]: 0.98 [0.72-1.32]) and other baseline covariates (HR [95% CI]: 0.93 [0.68-1.26]). No statistically significant associations were found between TTh and diabetes. For hyperlipidemia, TTh group presented statistically significant improvement on low-density lipoprotein and total cholesterol.

**Conclusion:**

TTh use among men with TD was not associated with increased CV risk in real-world clinical practice.

Testosterone is an important metabolic and vascular hormone for maintaining male physiology. Besides sexual dysfunction, testosterone deficiency (TD) increases risk for obesity, metabolic syndrome, type 2 diabetes mellitus (T2D), major adverse cardiovascular events (MACE), depression, Alzheimer disease, severe COVID-19, and increases the incidence of mortality [1-11]. In the United States, the prevalence of TD was 38.7% in men aged ≥45 years presenting to primary care offices in the Hypogonadism in Males study [12]. The prevalence increased to approximately 50% among individuals with diabetes or obesity [12]. TD and its related complications increased healthcare utilization and costs [13, 14]. Testosterone therapy (TTh) normalizes testosterone concentrations to a healthy level with the goal of reducing both hypogonadism symptoms and decreasing the risk of comorbidities [15]. Recent randomized controlled trials, including the Testosterone Trials, T4DM, and TRAVERSE, have provided evidence of the beneficial effects of TTh in older hypogonadal men on sexual function, muscle mass and strength, bone density, and the improvement of anemia. They also provided reassurance with regard to TTh and prostate safety [16-23].

There has been controversy regarding TTh and cardiovascular (CV) safety. In 2010, the fear that TTh would increase CV events in men was initiated by the Testosterone in Older Men with Mobility Limitations (TOM) Trial, in which 209 hypogonadal men with limitations in mobility and a high prevalence of chronic disease (mean age, 74 years) were assigned to receiving placebo or testosterone gel for 6 months [24]. The trial was stopped early, due to the higher frequency of self-reported, CV-related adverse events [25]. The study had multiple shortcomings [5]. Since then, studies have been mostly reassuring. A systematic review on TTh and CV risk found that TTh was not associated with increased CV mortality or morbidity [26]. In fact, a recent meta-analysis of 35 placebo-controlled TTh trials involving 5601 men with TD with a mean duration of treatment of 9.5 months found no increased risk of CV events between the TTh and placebo groups [27]. Still, no clinical trials of TTh published to that date had been adequately powered to assess CV events. However, a Canadian cohort study using claims data that compared 10 311 men treated with testosterone replacement therapy and 28 029 controls found that long-term exposure to testosterone was associated with reduced risk of CV events [28]. A Californian study using electronic health record (EHR) data drew the same conclusion as the Canadian study [29].

In this context, the TRAVERSE trial was important, as it was a large randomized controlled trial comparing long-term transdermal testosterone gel vs placebo gel in 5246 hypogonadal men, 45 to 80 years of age with preexisting or high risk of cardiovascular disease (CVD) [30]. The study concluded that among men with hypogonadism and established CVD or multiple risk factors, TTh is similar to placebo with respect to the occurrence of MACE. However, TRAVERSE was conducted in hypogonadal men using transdermal testosterone, and a large proportion of participants achieved a suboptimal level of plasma testosterone. Therefore, TRAVERSE may not be generalizable to the use of injectable testosterone and the achievement of higher testosterone concentrations.

In addition, TD is associated with insulin resistance, visceral obesity, dyslipidemia, and T2D, all of which exacerbate CV risk [31] that may be improved by TTh. Indeed, the T4DM trial showed decreased T2D risk in overweight/obese men, whose testosterone levels were frequently low, while TRAVERSE trials did not demonstrate the efficacy of TTh in preventing progression from prediabetes to diabetes or improving glycemic control [20, 32]. TTh also reduces total cholesterol, low-density lipoprotein (LDL) cholesterol, and triglycerides, while sometimes increasing high-density lipoprotein (HDL) cholesterol [33].

This study aimed to assess the association between CV safety and long-term TTh in men with TD in real-world settings—using data from the EHRs obtained through The National Patient-Centered Clinical Research Network (PCORnet) supported network. We also investigated the role of TTh on 2 important CV risk factors—diabetes and hyperlipidemia.

## Method

### Sample Selection and Cohort Creation

A total of 23 622 male patients with at least one diagnosis record of TD from January 1, 2012, to June 30, 2023, were extracted from 3 healthcare systems under the Research Action for Health Network (REACHnet), including Tulane Hospital, Ochsner Health System, and University Medical Center New Orleans. TD was defined by ICD-CM codes as primary due to testicular injury/surgery (ICD-9-CM: 257; ICD-10-CM: E29) or secondary to a pituitary problem (ICD-9-CM: 253; ICD-10-CM: E23). A serum testosterone level of less than 350 ng/d (12.1 nmol/liter) was used to confirm TD diagnoses [34]. Patients with confirmed testosterone deficiency were defined as patients with at least 2 TD diagnosis and low serum testosterone level. Only patients without malignancies were selected. With a 1-year washout period, adult patients with at least 6 months baseline and 1-year follow-up data for continuous enrollment were selected.

Patients were then divided into 2 cohorts based on their testosterone therapy (TTh) usage. An intention-to-treat approach was used for definitions of TTh users and non-TTh users. Patients were analyzed according to their initial exposure group, regardless of subsequent changes to their exposure status during follow-up. To examine the long-term impact of testosterone therapy, patients treated with continuous TTh for at least 1 year were defined as the TTh group. Patients who opted against TTh were identified as the non-TTh group. The TTh and non-TTh cohorts were matched 1:1 at baseline by the propensity score calculated by age, race, the Charlson Comorbidity Index (CCI), and continuous serum testosterone.

The study was approved by the institutional review board (IRB) and the requirements to obtain informed consent were waived following 45 CFR 46.116 (d). No ethical concerns were brought forth by the IRB.

### Descriptive and Statistical Analysis

Baseline characteristics were compared between the TTh and non-TTh groups. The baseline characteristics included demographic information, vital signs, laboratory results, and preexisting conditions, with a particular focus on conditions associated with CVD. Continuous variables were analyzed by 2-sample *t* tests and presented as mean and SD. Categorical variables were analyzed by the Chi-square test and presented as number and percentage proportions.

To assess CV risk, we used MACE, which was defined as a composite outcome of myocardial infarction, stroke, and cardiovascular death [35]. To assess differences in CV risk between the TTh group and non-TTh group regarding the development of CV events, we utilized Kaplan-Meier analysis by generating cumulative incidence graphs and conducting log-rank tests. Furthermore, Cox regressions were employed to quantify the CV risk among the TTh and non-TTh groups, presenting hazard ratios (HRs) and their corresponding 95% CIs. Several Cox regressions were tested, including an unadjusted model, a model adjusted by pre-CVD conditions, and models adjusted by pre-CVD conditions and covariates that displayed statistically significant differences in baseline characteristics comparisons. Hot deck imputation was applied to replace missing values with the observed response from the most “similar” unit [36].

Exploratory analyses were performed to investigate the TTh impact on diabetes and hyperlipidemia. For patients without diabetes or hyperlipidemia history, we examined disease incidence during follow-up via time-to-event analyses. For patients with diabetes or hyperlipidemia, we compared glycated hemoglobin (HbA1c) and lipid files at pre- and post-treatment periods by 2-sample paired *t* tests, respectively. Diabetes was defined by ICD-9/10 codes, HbA1c ≥ 6.5%, and/or antidiabetic medications. Hyperlipidemia was defined by ICD-9/10 codes, LDL ≥ 130 mg/dL, and/or statin medications. To robustly assess the long-term effect of TTh, we focused on patients with the condition who had at least one required laboratory test within 2 years before the index date and after 1 year of follow-up. For example, among patients with diabetes, at least one HbA1c test within 2 years before the start date and at least one HbA1c test after the 1 year following the follow-up date were required to compare the pre- and post-period of TTh.

## Results

**Figure 1.**
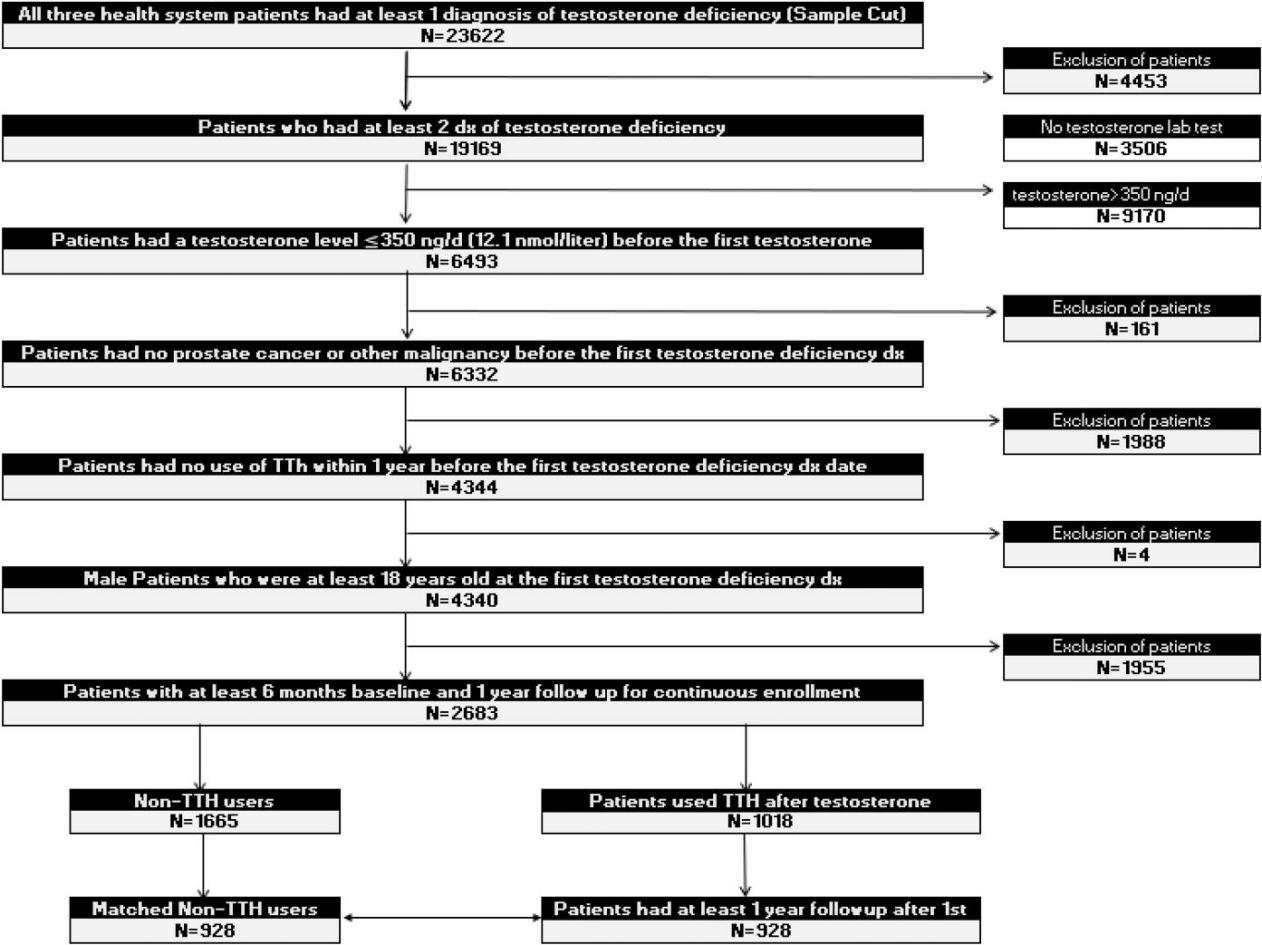
Sample selection patient flow chart. Selecting from 3 healthcare systems with a total of 23 622 patients, the final cohort of 2663 continuously enrolled adult male patients with confirmed TD diagnosis and without malignancy were identified. Two distinct groups emerged: 1018 patients who opted for TTh after TD diagnosis, and 1665 patients who opted against TTh (non-TTh group). A total of 928 patients who maintained a minimum one-year follow-up post-initial TTh prescription were selected as the TTh users. Another 928 patients as non-TTh users were matched 1:1 by propensity score matching on baseline age, race, CCI, and serum testosterone. *Non-TTh Users: Patients who opted against TTh. *TTh Users: Treated with continuous TTh for at least 1 year. *TTh Users and Non-TTh Users were 1:1 matched by baseline age, race, CCI, and serum testosterone.

Figure 1 presents the patient flow in the study. The final cohort of 2663 patients were selected from the 23 622 patients. Two distinct groups emerged: 1018 patients who opted for TTh after TD diagnosis, and 1665 patients who opted against TTh (non-TTh group). A total of 928 patients who maintained a minimum 1-year follow-up after initial TTh prescription was further examined to explore the long-term effects of TTh. To facilitate a comparison of CV risk over the follow-up period between TTh and non-TTh groups, 2 cohorts were achieved after propensity score matching.

The final sample included 928 patients (age 52.95 ± 12.38) in the TTh group with a median of 3 years of follow-up, and 928 patients (age 52.68 ± 13.21) in the non-TTh group with a median of 3 years of follow-up. Baseline characteristics for the 2 groups are outlined in Table 1. The majority of the patients were White (TTh: 74.25%; non-TTh: 74.35%). Hyperlipidemia was the most prevalent baseline underlying comorbidity (TTh: 69.07%; non-TTh: 67.82%), followed by hypertension (TTh: 64.98%; non-TTh: 59.91%), and diabetes (TTh: 33.30%; non-TTh: 29.31%). The 2 groups statistically differed in a few characteristics. The TTh group exhibited higher body mass index (BMI) and diastolic blood pressure (DBP) than the non-TTh group. Additionally, TTh group exhibited a higher prevalence of hyperlipidemia, hypertension, depression, and anxiety in comparison to their non-TTh counterparts. Although the above conditions were significantly different between the 2 groups, there were no statistically significant differences in the CV-related conditions in medical histories and testosterone-related laboratory results between the TTh group and the non-TTh group at baseline.

**Table 1.** Baseline characteristics

Characteristic	TTh users		Non-TTh users		*P* value
Age, years					
Age, years, mean (SD)	52.95	12.38	52.68	13.21	.6443
Age, years, median (IQR)	52.82	44.14-61.59	53.31	42.15-62.69	
Age ≥65 years, no. (%)	163	17.56%	173	18.64%	.5466
Race or ethnic group, no. (%)					
White	689	74.25%	690	74.35%	.9386
Black	184	19.83%	181	19.50%	
Other/Unknown	37	3.99%	36	3.88%	
Hispanic	18	1.94%	21	2.26%	
Charlson Comorbidity Index (CCI)					
CCI, mean (SD)	0.81	1.45	0.72	1.35	.1393
CCI, median (IQR)	0	0-1	0	0-1	
Vital signs					
**Body mass index (kg/m** ^2^ **), mean (SD)**	**34.23**	**7**.**66**	**33.35**	**7**.**44**	.**0138**
Body mass index (kg/m^2^), median (IQR)	33	29-38	32	28-37	
SBP (mmHg), mean (SD)	130.93	14.39	130.10	14.58	.2235
SBP (mmHg), median (IQR)	130	120-139	130	120-138	
**DBP (mmHg), mean (SD)**	**80.06**	**9**.**53**	**79.07**	**10**.**09**	.**0327**
DBP (mmHg), median (IQR)	80	74-86	80	72-86	
Lipid levels (mg/dL)					
HDL cholesterol, mean (SD)	42.09	11.19	42.39	10.37	.6125
HDL cholesterol, median (IQR)	41	34-47	41	36-49	
LDL cholesterol, mean (SD)	104.01	33.33	103.15	34.06	.6517
LDL cholesterol, median (IQR)	104.6	79.6-125.8	102.1	79.8-125.3	
Triglycerides, mean (SD)	162.37	170.26	150.02	102.16	.1120
Triglycerides, median (IQR)	134	94-188	123	88-182	
Total cholesterol, mean (SD)	176.77	39.79	175.06	39.55	.4412
Total cholesterol, median (IQR)	176	149-201	172	146-199	
Lab results					
Hematocrit (%), mean (SD)	43.61	4.37	43.47	4.09	.5340
Hematocrit (%), median (IQR)	43.9	40.7-46.6	43.8	41.1-46.1	
Prostate-specific antigen level (ng/mL), mean (SD)	1.27	1.77	1.58	2.58	.1933
Prostate-specific antigen level (ng/mL), median (IQR)	0.67	0.37-1.4	0.71	0.47-1.7	
Testosterone level (ng/dL), mean (SD)	271.44	275.88	283.19	242.07	.3699
Testosterone level (ng/dL), median (IQR)	227.50	128-313	272.00	151.5-354	
Preexisting conditions, no. (%)					
Myocardial infarction	28	3.02%	34	3.66%	.4383
Unstable angina	30	3.23%	27	2.91%	.6865
Stroke	34	3.66%	31	3.34%	.7048
Atrial fibrillation	50	5.39%	39	4.20%	.2321
Heart failure	59	6.36%	47	5.06%	.2300
Cerebrovascular disease	63	6.79%	59	6.36%	.7079
Ischemic heart disease	149	16.06%	140	15.09%	.5645
Peripheral artery disease	77	8.30%	68	7.33%	.4363
Chronic kidney disease	222	23.92%	223	24.03%	.9566
Diabetes*^a^*	309	33.30%	272	29.31%	.0640
Liver disease	97	10.45%	96	10.34%	.9394
**Hyperlipidemia** *^b^*	**641**	**69**.**07%**	**583**	**62**.**82%**	.**0045**
**Hypertension**	**603**	**64**.**98%**	**556**	**59**.**91%**	.**0243**
**Anxiety**	**274**	**29**.**53%**	**233**	**25**.**11%**	.**0327**
**Depression**	**210**	**22**.**63%**	**170**	**18**.**32%**	.**0214**
Smoker	13	1.40%	10	1.08%	.5291

Bold values indicated statistical significance (*P*-value <.05).

Abbreviations: DBP, diastolic blood pressure; HDL, high-density lipoprotein; IQR, interquartile range; LDL, low-density lipoprotein; SBP, systolic blood pressure; TTh, testosterone therapy.

^a^Diabetes was defined by ICD-CM codes, HbA1c ≥6.5% or antidiabetic medications.

^b^Hyperlipidemia was defined by ICD-CM codes, LDL ≥130 mg/dL or statin medications.

**Figure 2.**
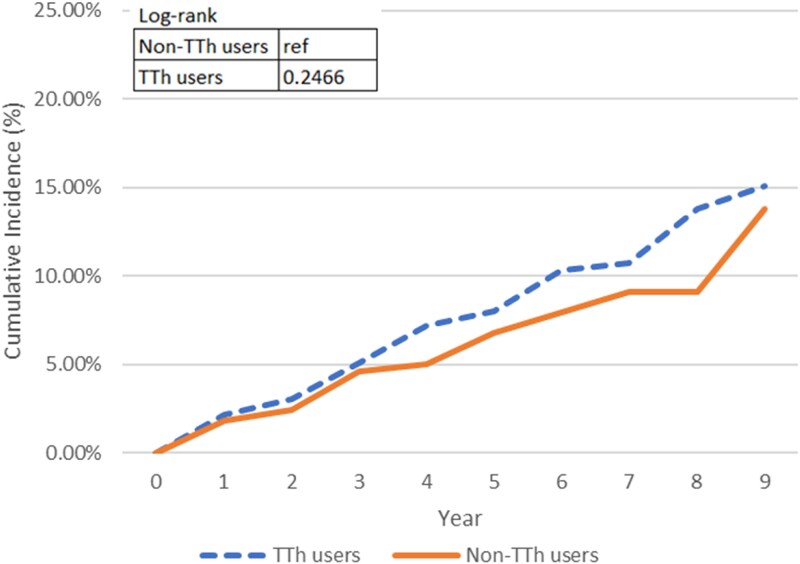
The dashed line represents TTh users, and the solid line represents non-TTh users. The log-rank test *P* value was .2466, indicating no statistically significant difference in MACE incidence between groups over time.

**Figure 3.**
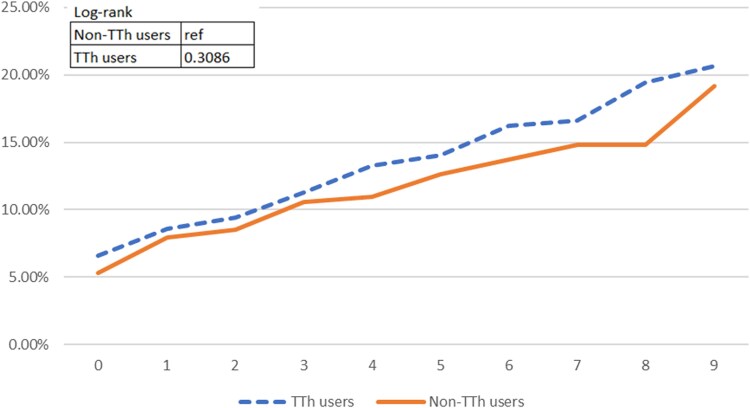
The dashed line represents TTh users and the solid line represents non-TTh users. The log-rank test *P* value was .3086, indicating no statistically significant difference in MACE incidence between groups over time after adjusting for MACE history at baseline.

Figures 2 and 3 present the Kaplan-Meier graphs on MACE. Among our study participants, we observed 867 TTh users without preexisting MACE conditions, of whom 61 experienced MACEs. Similarly, 870 non-TTh users without preexisting MACE conditions encountered 49 MACEs by the end of the study. A higher incidence probability was evident in TTh group in the third year of follow-up based on the cumulative incidence graph (Fig. 2). However, the cumulative incidence graph demonstrated that there is no statistically significant difference between the 2 groups on the MACE incidence development, with the *P* value of the log-rank test exceeding .05. This pattern persisted when considering preexisting MACE conditions (Fig. 3). A breakdown of MACE endpoints is presented in Table 2.

**Table 2.** MACE endpoint breakdown

Group	MI		Stroke		CVD death	
TTh users (N = 928)	36	3.02%	25	3.66%	0	0.00%
Non-TTh users (N = 928)	23	3.66%	23	3.34%	3	0.32%

Abbreviations: CVD, cardiovascular disease; MACE, major adverse cardiovascular events; MI, myocardial infarction; TTh, testosterone therapy.

Table 3 shows the results of 4 Cox regressions that assessed the impact of TTh on CV risk. The unadjusted model presented no statically significant association between TTh usage and CV risk (HR = 1.01; 95% CI, 0.75-1.36). After adjustment by preexisting MACE conditions, the regression results showed minor negative, statistically nonsignificant, association (HR = 0.98; 95% CI, 0.72-1.32). The model adjusted by preexisting MACE conditions and covariates that displayed statistically significant differences in baseline characteristics also presented a slight negative statistically nonsignificant association (HR = 0.93; 95% CI, 0.68-1.26). Missing values were observed in BMI and DBP. After applying hot deck imputation to replace missing values in BMI and DBP, the above model still displayed minor negative statistically nonsignificant association (HR = 0.95; 95% CI, 0.70-1.28). Therefore, across all models—unadjusted and adjusted, with and without imputations—we consistently found statistically nonsignificant differences between the TTh and non-TTh groups. Additionally, patients with preexisting MACE, hyperlipidemia, and/or hypertension conditions presented statistically significant greater CV risks. This highlighted the importance of CV management during the utilization of TTh, as populations with preconditions are continuously at high risk for the development and progression of CV events.

**Table 3.** Hazard ratio estimates

Model	Variables	HR (95% CI)	*P* value
Model 0	TTh users	1.01 (0.75, 1.36)	.947
			
Model 1	TTh users	0.98 (0.72, 1.32)	.88
	**Pre-CVD Conditions**	**15.91 (11.53, 21.94)**	**<.001**
			
Model 2 (Missing)	TTh users	0.93 (0.68, 1.26)	.633
	**Pre-CVD Conditions**	**12.42 (8.81, 17.50)**	**<.001**
	BMI	0.98 (0.96, 1.01)	.135
	DBP	0.99 (0.98, 1.01)	.274
	**Hyperlipidemia**	**1.70 (1.06, 2.72)**	**.028**
	**Hypertension**	**2.77 (1.67, 4.58)**	**<.001**
	Anxiety	0.73 (0.48, 1.10)	.138
	Depression	1.05 (0.69, 1.61)	.81
			
Model 3 (Imputation)	TTh users	0.95 (0.70, 1.28)	.715
	**Pre-CVD Conditions**	**12.28 (8.72, 17.30)**	**<.001**
	BMI	0.98 (0.96, 1.00)	.06
	DBP	0.99 (0.98, 1.01)	.41
	**Hyperlipidemia**	**1.68 (1.06, 2.68)**	**.028**
	**Hypertension**	**2.73 (1.67, 4.46)**	**<.001**
	Anxiety	0.73 (0.48, 1.09)	.126
	Depression	1.07 (0.71, 1.62)	.747

Bold values indicated statistical significance (*P*-value <.05).

Abbreviations: BMI, body mass index; CVD, cardiovascular disease; DBP, diastolic blood pressure; TTh, testosterone therapy.

**Figure 4.**
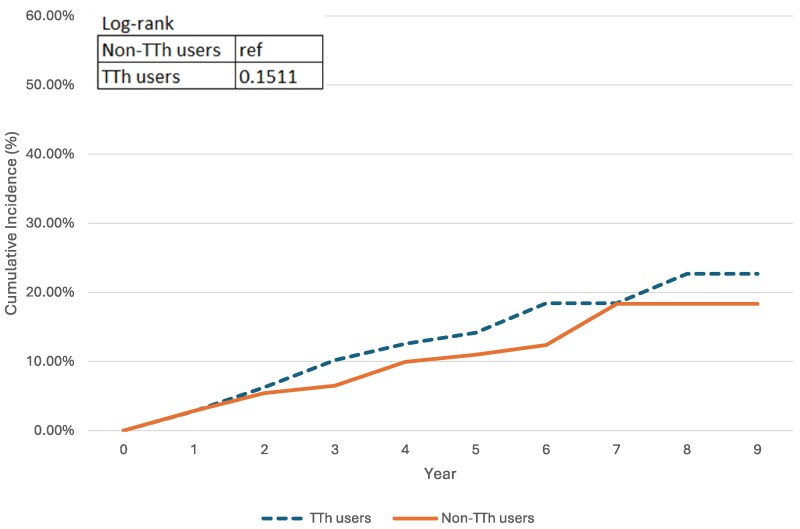
The dashed line represents TTh users, and the solid line represents non-TTh users. The log-rank test *P* value was .1511, indicating no statistically significant difference in diabetes incidence between groups over time.

**Figure 5.**
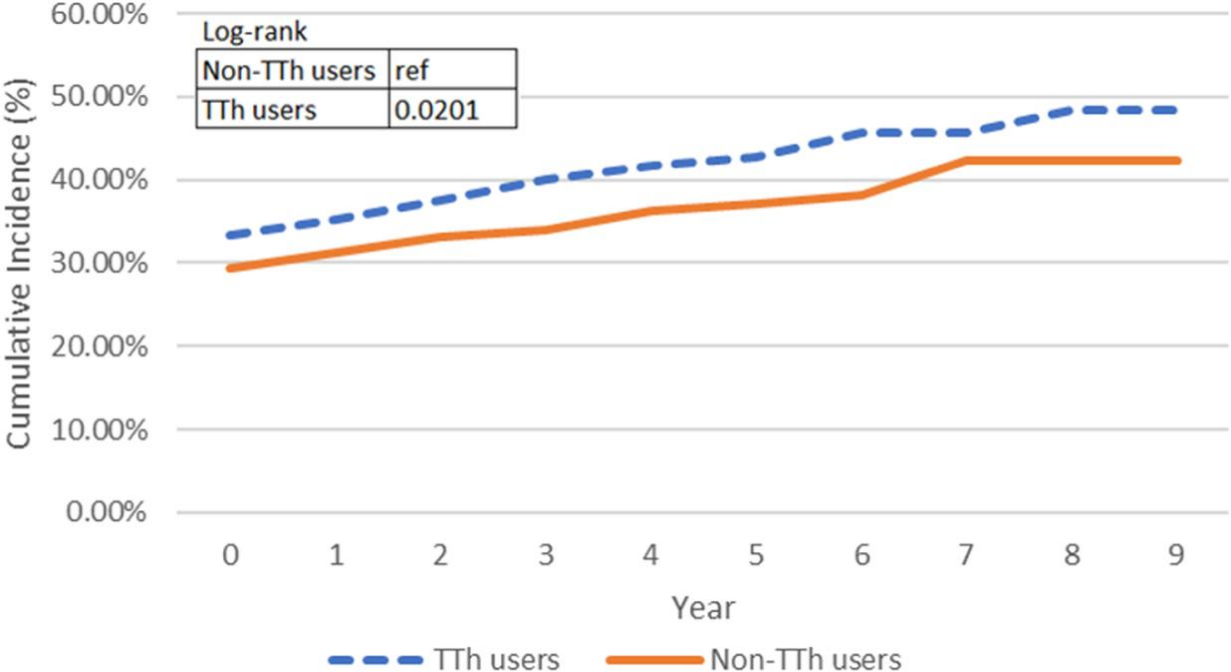
The dashed line represents TTh users and the solid line represents non-TTh users. The log-rank test *P* value was .0201, indicating statistically significant difference in diabetes incidence between groups over time after adjusting for diabetes history at baseline.

**Figure 6.**
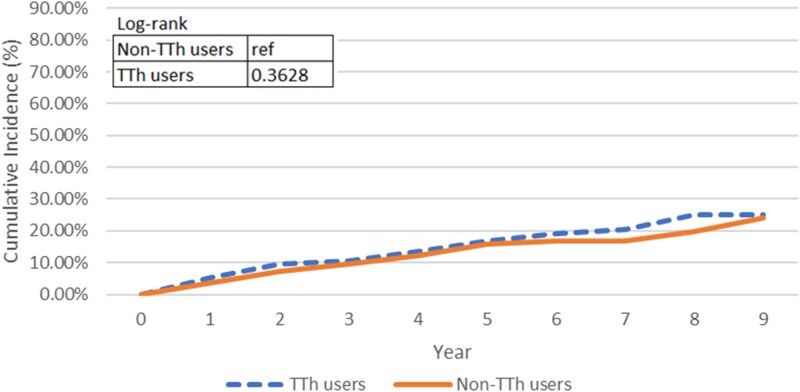
The dashed line represents TTh users, and the solid line represents non-TTh users. The log-rank test *P* value was .3628, indicating no statistically significant difference in hyperlipidemia incidence between groups over time.

**Figure 7.**
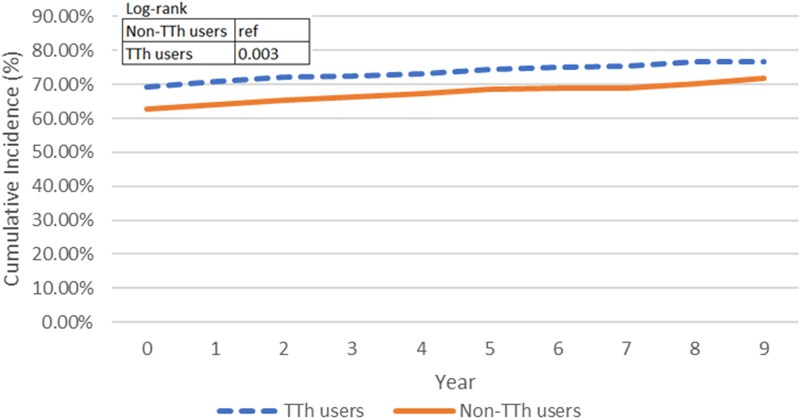
The dashed line represents TTh users and the solid line represents non-TTh users. The log-rank test *P* value was .0030, indicating no statistically significant difference in hyperlipidemia incidence between groups over time after adjusting for hyperlipidemia history at baseline.

Figure 4 graphs the cumulative incidence curves for patients without diabetes. Among the TTh and non-TTh groups, 619 and 656 patients without diabetes at baseline developed 75 and 63 new diabetes incidence during the study period. With a mean time to event of 7.92 years for TTh group and 6.29 years for non-TTh group, the cumulative incidence curves were not distinct from each other (*P* = .1511), indicating that the TTh group did not present higher diabetes incidence than the non-TTh group. When adjusted by the pre-baseline diabetes condition, with higher incidence in TTh group than non-TTh group, the cumulative incidence curves in Fig. 5 became statistically significant (*P* value = .0201); however, the significance suggested that the change might be due to the difference of the diabetes history distribution between the TTh and non-TTh users. Following the same algorithm, out of 632 patients without a history of hyperlipidemia, 81 developed new incidence of hyperlipidemia during follow-up, with 40 cases in the TTh group and 41 cases in the non-TTh group. The twisted cumulative incidence curves in Fig. 6 and log-rank test showed no statistically significant difference, indicating that the cumulative incidence of hyperlipidemia of TTh did not differ from non-TTh groups (*P* = .3628). As with the diabetes incidence exploration, when adjusted for hyperlipidemia history in Fig. 7, the TTh group had higher hyperlipidemia incidence, but the statistically significant difference (*P* =.0030) between the 2 groups may be explained by the different baseline hyperlipidemia distribution.

Table 4 demonstrated the change of HbA1c and lipid files due to TTh among patients with diabetes and hyperlipidemia, respectively. A total of 61 TTh users met the HbA1c test time requirement. The mean HbA1c remained around 7% before and after treatment, with no statistically significant difference observed. The 2-sample paired *t* test result of diabetes exploration suggested that the TTh does not affect diabetes development in this population. Similar to HbA1c in diabetes exploration, HDL, LDL, total cholesterol, and triglycerides were compared between the 2 groups before and after treatment. The HDL remained at 41 to 42 mg/dL for pre- and post-treatment among the 461 TTh users who satisfied the criteria. LDL, triglycerides, and total cholesterol decreased by approximately 5 mg/dL, respectively. Consistent with prior studies, only LDL (*P* = .002) and total cholesterol (*P* = .0025) exhibited statistically significant decreases.

**Table 4.** Pre- and post-treatment biomarkers comparison

Laboratory test	N	Median	IQR	Mean	Std	*P* value
HbA1c (%)						
Pre-treatment	61	7.1	6.5-8.1	7.47	1.51	.1765
Post-treatment		6.8	6.2-7.8	7.17	1.43	
HDL cholesterol						
Pre-treatment	461	40	34-47	42.05	11.26	.2737
Post-treatment		41	35-48	41.64	10.47	
**LDL cholesterol**						
Pre-treatment	447	105.2	76.4-130.8	105.15	35.29	.0002
Post-treatment		96.2	73.4-124.4	99.4	36.33	
Triglycerides						
Pre-treatment	461	142	98-194	173.87	198.39	.5255
Post-treatment		129	88-203	169.85	162.35	
**Total cholesterol**						
Pre-treatment	461	177	149-206	179.14	42.03	.0025
Post-treatment		173	143-200	173.56	41.82	

Laboratory measurements with statistical significance (*P*-value <.05) were bolded.

Abbreviations: HDL, high-density lipoprotein; IQR, interquartile range; LDL, low-density lipoprotein.

## Discussion

We compared MACE as the CV outcome between the TTh group and non-TTh group in a real-world clinical setting. The hazard ratios and the Kaplan-Meier analyses indicated that there is no statistically significant association between TTh and adverse CV events. The Kaplan-Meier curves showed the long-term TTh safety data on diabetes and hyperlipidemia incident development. The pre-TTh and post-TTh comparison among TTh users also suggested positive impacts of TTh on diabetes and hyperlipidemia progression.

The CV safety of TTh has been a topic of long-lasting controversy despite evidence that TTh provides no CV risk [5, 26, 27]. The TRAVERSE trial was performed to evaluate the CV risks/safety of TTh and also found no increased incidence of MACE compared to placebo [22, 30]. Consistent with these studies, our findings demonstrate no statistically significant association between TTh and CV risk. Some real-world studies conducted using claims data and EHR data demonstrated reduced risk of cardiovascular events under long-term exposure to testosterone therapy [28, 29].

A strength of our study is that 582 patients used TTh via an injection route (62.72% of the TTh users); only 317 patients (34.16%) used testosterone transdermal preparations (cream/gel/patch) and 29 patients (3.13%) took oral testosterone. In most of the previous studies, participants received transdermal testosterone. For example, the TRAVERSE trial involved participants receiving daily transdermal testosterone or placebo gel [22]. The transdermal route is characterized by poor absorption compared to injections. In our study, 62.72% of the TTh group used injectable testosterone who had a higher level of testosterone compared with those whose used noninjected forms/routes. This provides greater assurance regarding the safety of testosterone on potential CV risk. The high testosterone concentration enhanced the validity and robustness of our findings regarding the safety of testosterone on potential CV risk. Further study could investigate the impact of different forms/routes of TTh on the CV risk and compare results with previous research [29, 37].

Another strength of our study is the use of real-world evidence derived from EHRs. EHRs provide a comprehensive and longitudinal view of patient health data and the vast amount of data available reflects the real-world pattern of health care utilization, enhancing the generalizability of findings. With the evidence from EHRs analysis, our study demonstrated that TTh does not appear to increase CV risk and can be safely used in clinical practice, even among patients with moderately high CV risk.

There are limitations in our study. First, the study assumes that patients categorized as TTh users adhered to their prescribed treatment regimen for at least 1 year. However, actual adherence and compliance rates were not assessed, and discontinuation or irregular use of TTh could influence outcomes. Second, although our study had a longer median follow-up duration than clinical trials, CV events as longer-term outcomes may manifest over extended periods. Thus, shorter follow-up durations may not provide a comprehensive understanding of TTh impact. Further, despite trying our best to match the TTh group and non-TTh group based on age, race, comorbidities, and serum testosterone levels, CVD risk factors such as BMI and hyperlipidemia remained higher in the TTh group at baseline. However, we adjusted for these statistically significant baseline covariates in our regression models and the results still demonstrated CV safety over long-term TTh use. Additionally, residual confounding variables, such as lifestyle choices, socioeconomic status, the experience of the clinicians treating patients, and other unmeasured variables could influence cardiovascular outcomes and were not recorded in EHRs. These uncaptured factors may have an impact on the analysis results. Instead of patient-reported CV events, the events were all identified by the ICD-10-CM diagnosis codes. There is a potential risk of misclassification of event diagnosis by the clinician, and yet the misclassification applies equally to the treatment and control groups. We followed the definition of MACE in the ACCORD clinical trial and used MACE to measure CV risk; however, there are other important CV conditions, such as thromboembolic adverse events and cardiac rhythm-related events, that could be taken into account. Assessing the impact of TTh on specific CV events is warranted in future studies. Lastly, the data are not publicly available as we used patient-level data from 3 health systems.

## Conclusion

Our study indicates that long-term testosterone use in patients with TD is associated with CV safety in real-world clinical practice.

## Data Availability

Restrictions apply to the availability of some or all data generated or analyzed during this study to preserve patient confidentiality or because they were used under license. The corresponding author will on request detail the restrictions and any conditions under which access to some data may be provided.

## Abbreviations

BMI: body mass index
CCI: Charlson Comorbidity Index
CV: cardiovascular
CVD: cardiovascular disease
DBP: diastolic blood pressure
EHR: electronic health record
HbA1c: glycated hemoglobin
HDL: high-density lipoprotein
HR: hazard ratio
LDL: low-density lipoprotein
MACE: major adverse cardiovascular events
T2D: type 2 diabetes
TD: testosterone deficiency
TTh: testosterone therapy
